# Analysis of Extended Spectrum Beta Lactamase (ESBL) Genes of Non-Invasive ESBL Enterobacterales in Southeast Austria in 2017

**DOI:** 10.3390/antibiotics12010001

**Published:** 2022-12-20

**Authors:** Astrid H. Paulitsch-Fuchs, Nadine Melchior, Theresa Haitzmann, Theres Fingerhut, Gebhard Feierl, Rita Baumert, Clemens Kittinger, Gernot Zarfel

**Affiliations:** 1 Biomedical Science, School of Health Sciences and Social Work, Carinthia University of Applied Sciences, St. Veiterstraße 47, 9020 Klagenfurt, Austria; 2 Diagnostic and Research Center for Molecular BioMedicine, Medical University of Graz, Neue Stiftingtalstraße 6, 8010 Graz, Austria

**Keywords:** *Escherichia coli*, *Klebsiella* spp., epidemiology, antimicrobial resistances, ESBL, infection, third generation cephalosporins

## Abstract

Extended spectrum beta lactamases producing Enterobacteriaceae are a major player in the antibiotic resistance challenge. In general, the situation regarding antibiotic resistance in Austria is very good compared to many other countries. Perhaps this is why there is a lack of data on the distribution of ESBL genes in the clinical setting. The aim of this study was to collect data on ESBL genes from a larger sample of human non-invasive clinical isolates from one region in Austria. In total, 468 isolates from different sample materials isolated at the Medical University of Graz from 2017 were examined. The most frequent organisms were *Escherichia coli* and *Klebsiella pneumoniae*. Among the enzymes produced, CTX-M-15 was clearly dominant, exotic ESBLs were only represented by three *Proteus mirabilis* isolates harboring genes for VEB-6 and one *P. mirabilis* for CTX-M-2, respectively. Compared to other countries, the results are in line with the expectations. The data help to better classify the many studies from the non-clinical field in Austria and to shift the focus slightly away from the exotic results and sample sites.

## 1. Introduction

Extended spectrum beta lactamases (ESBLs), especially found in Enterobacteriaceae, are one of the big players in the rise in antibiotic resistance in Gram-negative bacilli. ESBLs are roughly defined as enzymes that lead to resistance to penicillins (at least) to third and/or fourth generation cephalosporins and aztreonam, and can be inhibited by beta-lactamase inhibitors. However, there are also variants that are insensitive to beta-lactamase inhibitors [1,2,3,4]. These were (and still are) evolved as a reaction to the introduction of modern cephalosporins. The first ESBLs could be observed in the enzyme family TEM and SHV, caused by mutations of already known non-ESBL variants in the 1980s [3,5]. In the following decades, new enzyme families with ESBL phenotype members have occurred. Nowadays, the CTX-M family is dominant worldwide with CTX-M-15 being the most often detected ESBL enzyme. While TEM ESBL has mostly disappeared, SHV ESBLs (close to the CTX-M family) are still one of the most common ESBLs found [5]. However, there are now dozens of other beta lactamase families that consist entirely of ESBL members, most of which belong to Ambler class A (like GES, VEB, BEL). However, some enzymes of Ambler class D (OXA-2 and OXA-10 derivates) also belong to the ESBL group of enzymes [1,3,5].

The successful spread of ESBL enzymes is also due to the high gene exchange within the Enterobacteriaceae and related groups like the Pseudomonadales. Many genes coding for ESBLs are found on mobile genetic elements (such as plasmids) and can be expressed in different species and genera. Therefore, ESBL enzymes are present in nearly all clinically important members of the Enterobacteriaceae family, with *Escherichia coli* and members of the genus *Klebsiella* being the most common isolates [6,7,8].

Bacteria that produce ESBL are by no means only found in the clinical setting. There are a number of studies that show that ESBL Enterobacteriaceae are colonizing the healthy normal population almost all around the world. These can be found in farm animals, pets and wildlife, on food, and in various habitats in the environment [8,9,10,11,12,13].

The studies from non-clinical areas are so numerous that sometimes a better picture of the situation outside the clinical setting (apart from the pure resistance data) is available. This is certainly true for Austria, for example, for the enzyme family GES, there is only evidence from water samples thus far [14,15,16,17,18,19].

The aim of this study is to provide an up-to-date overview of the predominant ESBL producers, enzymes, and co- resistance from human non-invasive isolates collected during one year (2017) as well as to be able to provide an up-to-date basis for the current studies from the non-clinical area.

## 2. Results

A total of 392 (83.76%) of the 468 isolates (from 423 patients, please see material and methods for details on the exclusion procedure) included in the analysis were *E. coli* followed by 54 (11.54%) *Klebsiella pneumoniae* isolates, and eight (1.71%) *Proteus mirabilis*. Other species were isolated five times or less often (Table 1). Isolates originated from various body sites can be summarized in six categories: the majority of isolates were isolated from the genito-urinary system (GUS; 343/468, 73.3%), followed by feces (39 isolates, 8.3%), wounds (34 isolates, 7.3%), skin (29 isolates, 6.2%), the upper (URT; 15 isolates 3.2%) and lower (LRT; eight isolates, 1.7%) respiratory tract.

All isolates were resistant to ampicillin (AM including the natural resistance of most isolated species) as well as to cefuroxime (CXM) and cefotaxime (CTX). Five isolates of *E. cloacae,* four *E. coli*, one *K. pneumoniae*, and one *P. mirabilis* (in total 1.3% of all isolates) showed resistance to meropenem (MEM), which is therefore the most effective drug available in in vitro testing (Table 2).

**Figure 1:**
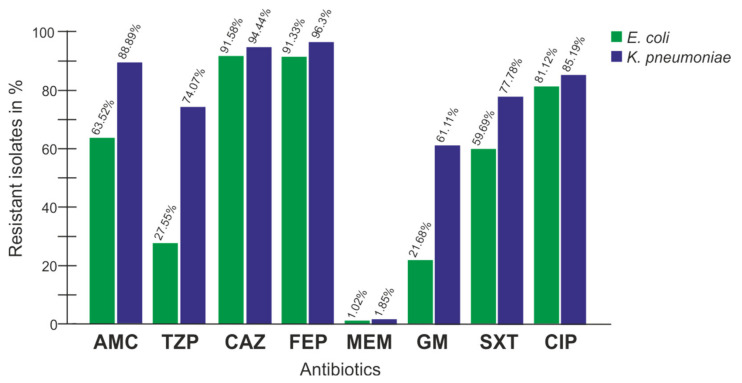
Resistance profile comparison of *E. coli* (n = 392) and *K. pneumoniae* (n = 54). AMC: amoxicillin/clavulanic acid; TZP: piperacillin/tazobactam; CAZ: ceftazidime; FEP: cefepime; MEM: meropenem; GM: gentamicin; SXT: trimethoprim/sulfamethoxazole; CIP: ciprofloxacin.

Highlighting the most important species *E. coli* and *K. pneumoniae* in comparison (Figure 1), the resistance patterns showed a high similarity with the exception of the ß-lactamase inhibitors and combinations (AMC, TZP) as well as GM. Generally, *E. coli* showed a lower percentage of resistance.

**Figure 2:**
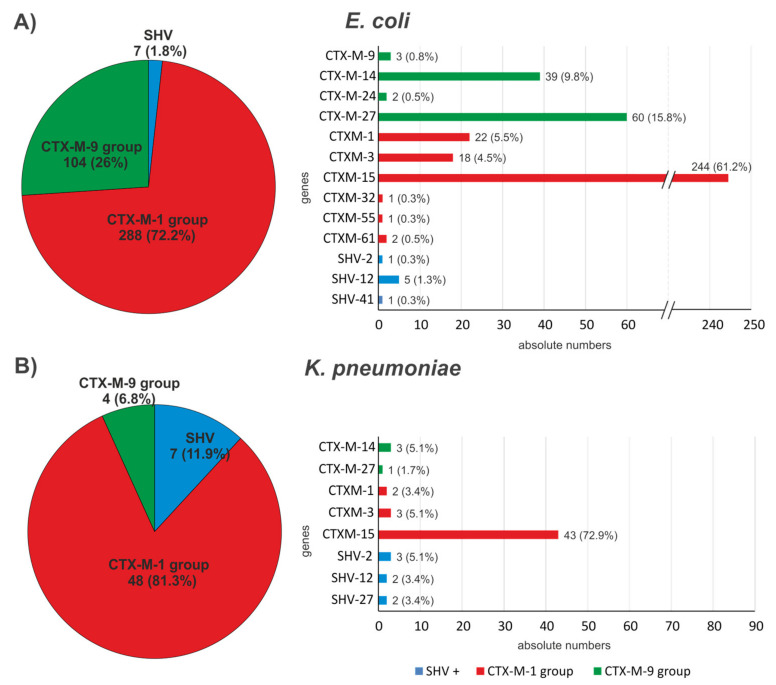
Bla genes coding for ESBL phenotypes isolated from (**A**) *E. coli* and (**B**) *K. pneumoniae* in absolute numbers and in (%). Left groupwise comparison; right genewise comparison.

A total of 399 ESBL genes and 148 non-ESBL variants (*bla*_TEM-1_, *bla*_SHV-1_, *bla*_SHV-11_; Table S1) were detected in the 392 *E. coli* isolates. The most abundant ESBL genes for *E. coli* were members of the *bla*_CTX-M-1 group_ (n = 288, 72.18%) (Figure 2). From the 54 *K. pneumoniae* isolates, 59 ESBL genes, and additionally, 87 non-ESBL variants (*bla*_TEM-1_, *bla*_SHV-1_, *bla*_SHV-11_, *bla*_SHV-28_, and *bla*_SHV-76_; Table S1) were detected. Forty-eight (81.36%) of the 59 ESBL genes belonged to the *bla*_CTX-M-1 group_. Not surprisingly, *bla*_CTX-M-15_ (a member of the CTX-M-1 group of genes) was proportionately most common in both species with 61.15% (n = 244) of the ESBL positive *E. coli* and 72.88% (n = 43) of the ESBL positive *K. pneumoniae*. ESBL *bla*_SHV_ gene variants were more abundant in the *K. pneumoniae* isolates (11.86%) than in the *E. coli* isolates (1.75%). For the *bla*_CTX-M-9 group_, the proportional abundance was higher in *E. coli* (26.07%) than in *K. pneumoniae* (6.78%).

CTX-M-15 was also the most widespread enzyme among the other species. *Bla*_VEB-6_ was detected three times (all in *P. mirabilis* isolates) and only one *bla*_CTX-M-2_ also in one *P. mirabilis* isolate was detected, none of those were carrying another ESBL gene (Table S1). There were eight *E. coli*, four *K. pneumoniae*, and one *E. cloacae*, each carrying two different ESBL genes (Table 3).

Genes for non-ESBL enzyme TEM-1 were also carried by a large proportion of isolates (across all species), whereas wild-type SHV, apart from two *E. coli* isolates, was other-wise only found in *K. pneumoniae* (belongs to the chromosomal wild-type of this species) (Table S1).

## 3. Discussion

The presence of ESBL-producing strains was documented in Austria for the first time in the 1990s. After the turn of the millennium, many more studies followed that documented and analyzed the presence of ESBL-carrying bacteria in Austria. Most of them focused on isolates from various livestock and wild animals as well as isolates from the environment. ESBL-producing Enterobacteriaceae could be detected in a wide variety of animals, but also on food and different areas of the water cycle [16,19,20,21,22,23,24,25].

However, there are few studies on the distribution of the various ESBL enzymes in the clinical setting. In detail, there are no studies dealing with Enterobacteriaceae species from these types of isolates. Epidemiological studies with large numbers of isolates are also available, but these are unfortunately limited to the phenotype [16,21,22,26].

The aim of the work was to capture the totality of the different ESBL genes at the present time for Austria, to document the occurrence of exotic variants, and thereby provide a basis for comparison for other (non-clinical) studies. Therefore, the limitations of the study are also predefined. The aim of this study was testing a high number of isolates for ESBL genes, strain backgrounds (via the species description) and genetic localization were not.

Due to the high occurrence in infections in general, the high proportion of *E. coli* and *Klebsiella* in the ESBL isolates is not surprising. The situation regarding the prevalence of ESBL producers, with about 10.3% of *E. coli* isolates and 10.5% for *Klebsiella pneumonia* isolates in 2017, is relatively moderate in Styria compared to other regions [1,27,28,29]

The low number of Enterobacter and Citrobacter in the isolates is somewhat critical. Here, the selection by intrinsic AmpC could have led to a false designation as AmpC hyperproducers, even if an ESBL was the cause of the resistance. Furthermore, it needs to be considered that the prevalence of the ESBL of Enterobacter is also low in other parts of Austria [22,30]. The dominance of the CTX-M-1 group and the CTX-M-15 enzyme, in particular, was not surprising. A comparison with Eisner et al. is a good illustration of the development in Austria over the last 20 years, which is in line with the global situation. Around the turn of the millennium, Eisner et al. found that about one third of the genes in the ESBLs studied encoded for a member of the CTX-M family; now this figure is well over 90%. CTX-M enzymes are dominating the ESBL occurrence worldwide [31,32,33]. Exotic ESBL enzymes, on the other hand, were very rare in the current study. Although there have been reports of CTX-M-2 (group) or GES ESBL being found in Austria in the recent past, none of the isolates examined were found to harbor genes encoding GES enzymes, and only one *P. mirabilis* harbored a CTX-M-2.

Furthermore, three *P. mirabilis* isolates carried a gene for VEB-6. These isolates have already been described in a case report, and originate from only one patient. This work was primarily concerned with the carbapenemases. However, since ESBL genes were also present in the isolates, these isolates were also included in this study [19,34].

Looking at the co-resistance, as expected, ciprofloxacin is a non-beta lactam-antibiotic with the highest resistance rates. The co-occurrence of ESBL (especially CTX-M-15 mediated) and fluoroquinolone resistance is a well-known phenomenon [5,35,36]. Resistance to the last line antibiotic meropenem is below 2% in *E. coli* and *Klebsiella* spp. This approximately corresponds to what is found in neighboring countries [28,29,37].

In most cases, ESBL enzymes are sensitive to beta-lactamase inhibitors. However, a high percentage of the isolates examined are resistant to amoxicillin-clavulanic acid, and the proportion of isolates resistant to piperacillin/tazobactam is also in the mid-range for most species, with the high proportion for *K. pneumonia* being particularly worrying. However, this is most likely not caused by inhibitors of resistant varieties of ESBL; these isolates carry other mechanisms that are likely to give them this additional resistance [3,38,39].

## 4. Conclusions

The clinical ESBL picture in Austria, especially in the federal state of Styria, shows that it is primarily caused by *E. coli* and *Klebsiella;* mainly by the CTX-M-15 enzyme. With the help of the non-invasive isolates analyzed, a good picture can be presented that allows for a better classification of the many studies on ESBL from animals, from the environment, or from individual patients.

## 5. Materials and Methods

### 5.1. Collection of Isolates

Between 1 January 2017 and 31 December 2017, all presumptive ESBL Enterobacteriaceae first isolates (*n* = 1057) from non-invasive infections were collected at the Diagnostic and Research Center for Molecular BioMedicine of the Medical University of Graz and stored at −80 °C in cryotubes (Viabank™, MWE Medical Wire, Corsham, UK). Isolates from every other month (January, March, May, July, September, November), in total 593 isolates, were tested for their ESBL genotype [40]. Different ESBL harboring species from one patient were treated as separated first isolates. From those double (or multiple) isolates from one patient were excluded/included in the statistical analysis following this procedure: All isolates from the same patient belonged to the same species and showed the same antibiotic resistance profile were only listed once (independent if isolated from the same or different body sites). Different ESBL species from the same patient are listed as such. Isolates that did not show any genetic ESBL profile were also not included in the statistical analysis, eight isolates fell into this category. Species and resistance patterns of these isolates are given in Table S2. Following this procedure, a total of 468 different isolates (101 from hospitalized patients and 367 from not hospitalized patients) were finally included in the statistical analysis. These isolates stem from a total of 423 patients as 23 patients were listed twice (14 not hospitalized, five hospitalized, and four were listed once in both groups) three patients three times (all not hospitalized), and two patients (one not hospitalized and one hospitalized) had four infections with different ESBL isolates.

### 5.2. Antibiotic Susceptibility Test

The isolates were characterized for their resistance pattern to 11 antibiotics by agar diffusion susceptibility testing according to the European Committee on Antimicrobial Susceptibility Testing (EUCAST) with ampicillin 10 μg (AM), amoxicillin/clavulanic acid 20 μg/10 μg (AMC), piperacillin/tazobactam 100 μg/10 μg (TZP), cefuroxime 30 μg (CXM), cefotaxime 5 μg (CTX), ceftazidime 10 μg (CAZ), cefepime 30 μg (FEP), meropenem 10 μg (MEM), gentamicin 10 μg (GM), trimethoprim/sulfamethoxazole 1.25 μg/23.75 μg (SXT), and ciprofloxacin 5 μg (CIP) (all with BD BBLTM Sensi-DiscTM paper discs; BD, Sparks, MD, USA). *E. coli* 25299 was used as the reference strain. The inhibition zone diameters were interpreted according to EUCAST guidelines [41].

### 5.3. Strain Cultivation

The samples were stored at –80 °C in cryotubes (Viabank™, MWE Medical Wire) until analysis. For MALDI-TOF identification and DNA isolation, strains were recultivated on Luria-Bertani (LB) agar plates (10 g/L tryptone, 10 g/L NaCl, 5 g/L yeast extract, 15 g Agar; all Carl Roth, Karlsruhe, Germany). For each isolate, one beat from the corresponding cryotube was plated on one LB-plate and incubated at 37 °C for 16 h.

### 5.4. MALDI-TOF

The exact identification of the bacteria was carried out using MALDI-TOF microflexTM LT/SH (Bruker AXS GmbH, Billerica, MA, USA) at the Institute for Laboratory Diagnostics and Microbiology of the Klinikum Klagenfurt am Wörthersee, Austria. The matrix used was the IVD matrix HCCA-portioned from Bruker.

### 5.5. DNA Isolation

A distinct colony was stirred into 50 μL of nuclease-free water and heated for 10 min at 100 °C on the heating block. Subsequently, centrifugation was carried out at 21,200 g for 2 min. The supernatant was transferred to a fresh reaction tube and kept in the freezer at −20 °C until further analysis.

### 5.6. Determination of ESBL Genes

PCR detection and gene identification were performed for different β-lactamase gene families, *bla*_CTX-M-1-group_*, bla*_CTX-M-2-group_, *bla*_CTX-M-9-group_, *bla*_GES_, *bla*_SHV_, *bla*_TEM_, and *bla*_VEB_. PCR and sequencing procedures were performed as described previously and carried out for all isolates that showed an ESBL-positive phenotype [42,43,44,45,46,47].

Briefly, for PCR, the Taq * 5x Master Mix (NEB, Ipswich, MA, USA) was used and the PCRs employing the different primer sets (Table 4) were performed on a GeneTouch Thermal Cycler (Biozym, Oldendorf, Germany) initializing with 94 °C for 5 min, 35 cycles at 95 °C for 30 s, 52 °C for 45 s, and 72 °C for 60 s, and closing with a final incubation for 10 min at 72 °C.

Gel electrophoresis was performed to identify positive PCR results, and positive PCR samples where purified using the Monarch PCR and DNA Cleanup Kit (NEB, Ipswich, MA, USA). The purified samples were sent for Sanger Sequencing (Eurofins, Luxemburg, Luxemburg or Genewiz, Leipzig, Germany). For this purpose, the corresponding reverse primer and the sample were pipetted into a 96-well PCR plate or in reaction tubes in the ratio requested by the respective company, sealed, and shipped.

Identification of the sequencing results was obtained using the BLAST (Basic Local Alignment Search Tool) program “Standard Nucleotide Blast” of the NCBI (National Center for Biotechnology Information, Bethesda, MD, USA).

### 5.7. Data Analysis

All data were analyzed using MS Excel (Office 2019) and the figures were prepared using MS Excel and CorelDraw X5 (Corel Corporation, Ottawa, ON, Canada, 2010, version 15.0.0.486).

## Figures and Tables

**Table 1:** Overview of the body site origins of the isolates.

	Isolates/Body Site (%)					
Species (n)	GUS	Feces	Wound	Skin	URT	LRT
*E. coli* (392)	309 (78.8%)	21 (5.4%)	27 (6.9%)	22 (5.6%)	9 (2.3%)	4 (1.02%)
*K. pneumoniae* (54)	28 (51.9%)	9 (16.4%)	2 (3.7%)	7 (13%)	5 (9.26%)	3 (5.56%)
*P. mirabilis* (8)	3 (37.5%)	1 (12.5%)	3 (37.5%)	-	1 (12.5%)	-
*E. cloacae* (5)	1 (20%)	2 (40%)	1 (20%)	-	-	1(20%)
*K. oxytoca* (5)	-	5 (100%)	-	-	-	-
*C. braakii* (1)	1 (100%)	-	-	-	-	-
*C. freundii* (1)	-	-	1 (100%)	-	-	-
*C. koseri* (1)	1 (100%)	-	-	-	-	-
*Salmonella* spp. * (1)	-	1 (100%)	-	-	-	-
sum (468)	343 (73.3%)	39 (8.3%)	34 (7.3%)	29 (6.2%)	15 (3.2%)	8 (1.7%)

GUS: genito-urinary system; URT: upper respiratory tract; LRT: lower respiratory tract; * no species identification with MALDI was possible.

**Table 2:** Percent of isolates included in the statistical analysis showing resistance to the tested antibiotics.

	AM	AMC	TZP	CXM	CTX	CAZ	FEP	MEM	GM	SXT	CIP
*E. coli*	100	63.5	27.6	100	100	91.6	91.3	1.02	21.7	59.7	81.1
*K. pneumoniae*	100	88.9	74.1	100	100	94	96.3	1.85	61	77.8	85.19
*P. mirabilis*	100	37.5	12.5	100	100	87.5	100	12.5	62.5	75	87.5
*E. cloacae*	100	100	20	100	100	100	100	100	80	80	80
*K. oxytoca*	100	80	0	100	100	20	100	0	100	80	0
*C. braakii*	100	100	100	100	100	100	0	0	100	0	100
*C. freundii*	100	100	100	100	100	100	100	0	100	0	0
*C. koseri*	100	0	0	100	100	100	100	0	0	100	0
*Salmonella* spp.	100	100	0	100	100	100	0	0	100	0	100
sum	100	66.7	32.5	100	100	91.2	91.9	1.28	28.9	62.2	80.6

AM: ampicillin; AMC: amoxicillin/clavulanic acid; TZP: piperacillin/tazobactam; CXM: cefuroxime; CTX: cefotaxime; CAZ: ceftazidime; FEP: cefepime; MEM: meropenem; GM: gentamicin; SXT: trimethoprim/sulfamethoxazole; CIP: ciprofloxacin.

**Table 3:** ESBL coding genes detected and the number of isolates with those genes (n).

Species	CTX-M-1 Group	CTX-M-2 Group	CTX-M-9 Group	SHV	VEB
*E. coli* *	*bla*_CTX-M-1_ (22) *bla*_CTX-M-3_ (18) *bla*_CTX-M-15_ (244) *bla*_CTX-M-32_ (1) *bla*_CTX-M-55_ (1) *bla*_CTX-M-61_ (2)	-	*bla*_CTX-M-9_ (3) *bla*_CTX-M-14_ (39) *bla*_CTX-M-24_ (2) *bla*_CTX-M-27_ (60)	*bla*_SHV-2_ (1) *bla*_SHV-12_ (5) *bla*_SHV-41_ (1)	-
*K. pneumoniae* **	*bla*_CTX-M-1_ (2) *bla*_CTX-M-3_ (3) *bla*_CTX-M-15_ (43)	-	*bla*_CTX-M-14_ (3) *bla*_CTX-M-27_ (1)	*bla*_SHV-2_ (3) *bla*_SHV-12_ (2) *bla*_SHV-27_ (2)	-
*P. mirabilis*	*bla*_CTX-M-15_ (3)	*bla*_CTX-M-2_ (1)	*bla*_CTX-M-27_ (1)	-	*bla*_VEB-6_ (3)
*E. cloacae* ***	*bla*_CTX-M-15_ (4)	-	*bla*_CTX-M-9_ (1)	*bla*_SHV-12_ (1)	-
*K. oxytoca*	*bla*_CTX-M-3_ (5)	-	-	-	-
*C. braakii*	-	-	-	*bla*_SHV-12_ (1)	-
*C. freundii*	*bla*_CTX-M-15_ (1)	-	-	-	-
*C. koseri*	*bla*_CTX-M-15_ (1)	-	-	-	-
*Salmonella* spp.	-	-	-	*bla*_SHV-12_ (1)	-

double positive isolates: * four with *bla*_CTX-M-15_ and *bla*_CTX-M-14_; two with *bla*_CTX-M-15_ and *bla*_CTX-M-27_; one with *bla*_CTX-M-15_ and *bla*_CTX-M-9_; one with *bla*_SHV-41_ and *bla*_CTX-M-15_. ** two with *bla*_SHV-27_ and *bla*_CTX-M-15_; one with *bla*_SHV-12_ and *bla*_CTX-M-15_; one with *bla*_CTX-M-15_ and *bla*_CTX-M-14._ ***one with *bla*_SHV-12_ and *bla*_CTX-M-9_.

**Table 4:** Primers used for PCR.

Gene Families	Primer	Sequence 5’ → 3’	Reference
*bla*_CTX-M-1-group_	f	TCTTCCAGAATAAGGAATCCC	[42]
	r	CCGTTTCCGCTATTACAAAC	
*bla*_CTX-M-2-group_	f	ATGATGACTCAGAGCATTCG	[43]
	r	TGGGTTACGATTTTCGCCGC	
*bla*_CTX-M-9-group_	f	ATGGTGACAAAGAGAGTGCA	[44]
	r	CCCTTCGGCGATGATTCTC	
*bla*_GES_	f	ATGCGCTTCATTCACGC C	[45]
	r	CTATTTGTCCGTGCTCAGG	
*bla*_SHV_	f	TGGTTATGCGTTATATTCGCC	[46]
	r	GGTTAGCGTTGCCAGTGCT	
*bla*_TEM_	f	TCCGCTCATGAGACAATAACC	[42]
	r	TTGGTCTGACAGTTACCAATGC	
*bla*_VEB_	f	GATAGGAGTACAGACATATG	[47]
	r	TTTATTCAAATAGTAATTCCACG	

bla: beta-lactamase; f: forward, r: reverse.

## Data Availability

The data can be obtained from the corresponding author upon reasonable request.

## Supplementary Materials

The following supporting information can be downloaded at: https://www.mdpi.com/article/10.3390/antibiotics12010001/s1, Table S1: Abundance of non-ESBL phenotype *bla* genes.; Table S2: Isolates with the ESBL phenotype but no detected ESBL genes.
