# Supplementary material for: Analysis of Extended Spectrum Beta Lactamase (ESBL) Genes of Non-Invasive ESBL Enterobacterales in Southeast Austria in 2017
Source: Antibiotics (Basel). 2022 Dec 20;12(1):1. doi: 10.3390/antibiotics12010001 (PMC9854808; doi:10.3390/antibiotics12010001)
Supplement: Supplementary file 1 [file antibiotics-12-00001-s001.zip › antibiotics-2094007-supplementary.pdf]

Table S1. Abundance of non-ESBL phenotype bla genes.

	bla $_{TEM-1}$	***bla*** $_{SHV-1}$	***bla*** $_{SHV-11}$	***bla*** $_{SHV-28}$	***bla*** $_{SHV-76}$
E. coli	146	1	1	-	-
K. pneumoniae	40	28	12	1	6
P. mirabilis	5	-	-	-	-
E. cloacae	4	-	-	-	-
K. oxytoca	5	-	-	-	-
C. braakii	1	-	-	-	-
C. freundii	1	-	-	-	-
S. spp	1	-	-	-	-
sum	203	29	13	1	6

Table S2. Isolates with ESBL phenotype but no detected ESBL genes.

Isolates	**non-ESBL genes**	**AM**	**AMC**	**CXM**	**CTX**	**GM**	**TZP**	**CIP**	**SXT**	**MEM**	**FEP**	**CAZ**
K. pneumoniae	***bla*** $_{SHV-1}$	R	S	R	R	S	S	S	R	S	S	R
E. coli	-	R	S	R	R	S	S	S	S	S	S	S
Providencia rettgeri	-	R	R	R	R	R	S	R	R	S	R	R
E. coli	-	R	R	R	R	R	R	R	R	S	R	R
E. coli	***bla*** $_{TEM-1}$, ***bla*** $_{SHV-11}$	R	R	R	R	S	R	R	R	S	R	R
K. variicola	***bla*** $_{SHV-11}$	R	R	R	R	S	R	S	S	S	R	R
K. oxytoca	-	R	R	R	R	S	R	S	S	S	R	R
C. freundii	***bla*** $_{TEM-1}$	R	R	S	-	S	S	S	S	R	S	S
